# Integration of a neuronal RNAseq dataset with the draft *Gryllus bimaculatus* transcriptome refines gene predictions and highlights potential systematic response to injury

**DOI:** 10.1371/journal.pone.0347755

**Published:** 2026-04-29

**Authors:** Felicia F. Wang, Harrison P. Fisher, Lisa M. Ledwidge, Joel H. Graber, Riley A. Grindle, Jarod A. Rollins, Hadley W. Horch

**Affiliations:** 1 Department of Biology, Bowdoin College, College Station, Brunswick, Maine, United States of America; 2 Mount Desert Island Biological Laboratory, Bar Harbor, Maine, United States of America; 3 The Jackson Laboratory, Bar Harbor, Maine, United States of America; University of Missouri Columbia, UNITED STATES OF AMERICA

## Abstract

The cricket *Gryllus bimaculatus* presents a compelling model for investigating neuroplasticity due to its unusual capability of adult structural reorganization. The molecular pathways underlying these changes remain poorly understood. Here, we reanalyzed RNAseq data, originally collected from deafferented and control prothoracic ganglia one, three, and seven days post-injury, which had previously been used to assemble a *de novo* transcriptome. In this current analysis, we aligned our original RNAseq data to the publicly available *G. bimaculatus* draft genome, and used the resulting alignments to refine and update the existing annotations. The integration added 3,868 novel genes, 9,172 new transcript isoforms including both protein-coding and putative non-coding transcripts, reflecting the likely regulatory importance of long non-coding RNAs in this neuronal context. These updated annotations were used as the basis for a DESeq2 differential expression analysis and subsequent functional enrichment analysis to further explore the potential molecular basis of this compensatory anatomical plasticity. Days one and three showed the largest post-deafferentation expression differences. Overall, more transcripts were upregulated rather than downregulated. Protein-protein associations enriched for GTPase-related signaling, hormone metabolism, and membrane dynamics were evident. We also identified a surprising enrichment of gene ontology terms related to muscle contraction in this neuronal-specific transcriptome. Toll receptor signaling emerged as a candidate pathway warranting further investigations. Our results demonstrate the importance of updating the reference transcriptome for analysis of highly specialized tissues or conditions, and serve as a resource for generating testable hypotheses about the well-conserved molecular mechanisms that may underlie this unique example of adult structural plasticity in the cricket.

## Background

Most adult organisms, especially mammals, are limited in their capacity to recover from neurological damage [1,2]. The Mediterranean field cricket, *Gryllus bimaculatus*, provides a model of neuroplasticity due to its demonstrated ability to compensate for neuronal damage with novel dendritic growth and synapse formation, even into adulthood. Specifically, the central auditory system, much of which resides in the prothoracic ganglion, reorganizes in response to deafferentation caused by unilateral transection of auditory afferents in the adult [3,4].

In crickets, auditory information is transduced by the auditory organs, located on the prothoracic limbs. Auditory afferents receive the sensory stimuli and convey this information into the prothoracic ganglion where they form synapses with several different auditory neurons [5,6]. These neurons exist as mirror image pairs and their dendritic arbors remain localized ipsilateral to the auditory input, typically not projecting contralaterally across the midline [7]. However, previous research has shown that after amputation of the prothoracic leg, which removes the auditory organ and severs the afferents, the deafferented dendrites of the ipsilateral auditory neurons sprout across the midline and form functional synapses with the intact auditory afferents on the contralateral side. This reorganization is evident whether deafferentation occurs in nymphs [8,9] or adults [3,10]. Various aspects of the physiological consequences of this compensatory behavior have been studied, however little is known about the molecular pathways and mechanisms underlying this growth.

Various *de novo* transcriptomes have been created for use in *Gryllus bimaculatus* [11–14], including one built with RNA from individual prothoracic ganglia of both control and deafferented adult male crickets [12]. Initially, this *de novo* transcriptome assembly was mined for the presence of developmental guidance molecules, though no differential analysis was completed [12].

We hypothesized that the annotated transcriptome from the draft genome, which was derived primarily from cricket embryonic and adult gonadal tissue [15], could be missing genes or transcript isoforms that could be found in a deeply sequenced, highly differentiated neuronal tissue [12] under specific conditions (deafferentation). Accordingly, a key goal of this current analysis was to first update the transcriptome by integrating novel transcripts and genes supported by the tissue-specific RNA-sequence data. We explicitly chose not to restrict novel transcripts or genes to those that were protein-coding, an inclusive approach that was motivated by evidence that non-coding RNAs, including long non-coding RNA’s (lncRNAs), are differentially expressed in neuronal tissues and may play important regulatory roles in normal and diseased neuronal systems [16,17].

In this updated analysis, we aligned the cricket prothoracic ganglia RNA-seq reads [12] to the publicly available *Gryllus bimaculatus* genome [15], predicted new transcripts, integrated a filtered set of these alignments into the reference transcriptome, and finally used the resulting updated transcriptome to quantify expression across these experimental samples. We then identified differential transcript/gene abundances one, three, and seven days post-deafferentation, and analyzed the resulting lists for functional enrichment to determine the types of transcripts that were differentially regulated over the course of the injury response. We discovered gene expression changes evident over the course of the compensatory growth response, allowing for the development of future hypotheses focused on pathways or key molecules critical to this process.

## Results and discussion

### Transcriptome assembly and analysis

This transcriptomic study focused on the cricket, *Gryllus bimaculatus*, whose nervous system has been shown to have an unusual level of adult structural plasticity [3,4,10]. We deafferented central sensory neurons, including the auditory neurons, in the prothoracic ganglion of the adult cricket by unilateral amputation of the prothoracic leg at the femur. The auditory organ resides just distal to the tibial-femoral joint on the prothoracic leg. Control amputations, designed to control for the stress of injury, consisted of removal of the distal tip of the tarsus. We harvested prothoracic ganglia one-, three-, and seven-days post-amputation. These time points were designed to capture transcriptional changes in response to the loss of activity (day one), during initial sprouting (days one and three), growth across the midline (days three and seven), and novel synapse formation (days three and seven; [3,18]). Although a *de novo* assembly from *G. bimaculatus* prothoracic ganglia was completed previously [12], the present study aligned the sequence reads to the published genome [15], generating updated transcriptome annotations, which were then used for differential expression analysis.

This genome-based analysis yielded 43,394 predicted transcripts from 20,533 genes (.gtf.fa files available at https://doi.org/10.7910/DVN/2BIYNK), which was far lower than the number predicted in our *de novo* assembly (374,383 transcripts; Fisher et al. 2018). This updated transcriptome yielded 4,452 tRNA genes and 4,453 tRNA transcripts, all of which had been previously annotated in the GBI transcriptome. In addition, it identified an increase in the number of annotations over the original draft genome assembly [15], which represented 28,529 transcripts from 17,871 genes (Table 1). Also, in comparison with the original genome assembly, the average and median transcript length increased from 2,624 and 1,848 nucleotides to 2,976 and 2,120 nucleotides, respectively, and the maximum transcript length increased from 27,129–62,365 nucleotides (Table 1).

**Table 1:** Summative detail from the original genome assembly (GBI) compared to our current, updated assembly (GBIG).

	GBI	GBIG (update)
N(Genes)	17,871	20,533
N(Transcripts)	28,529	43,394
Average transcripts per gene	1.6	2.1
Max transcripts per gene	19	22
Average transcript length	2,624	2,976
Median transcript length	1,848	2,120
Max transcript length	27,129	62,365

In cases where our assembly suggested new transcript isoforms to existing GBI-annotated genes, we preserved the GBI name and annotation, but added the isoforms to the gene definition. In total, 9,172 new transcript isoforms were added to 6,115 genes. Our updated assembly also resulted in 885 instances where the StringTie evidence suggested that neighboring regions on the draft genome, which were originally annotated as separate genes, are instead separate components of a single transcription unit. In such cases, our algorithm gave a new GBIG identifier to the complete gene, while retaining the transcript identifiers from the existing subunits from the GBI assembly. Table 2 shows the distribution of the number of genes joined together by our evidence, while the S1 Table gives a complete annotation of all genes, including both the new identifier along with the GBI genes that were joined.

**Table 2:** Evidence-based merging of GBI gene neighbors. The revised GBIG transcriptome included multiple instances of evidence that neighboring GBI-annotated genes form a single transcriptional unit. Combinations ranged from two neighbor regions, which occurred 677 times, up to seven neighbor regions, which occurred twice.

# of joined GBI neighbors	# of GBIG Instances
2	677
3	140
4	34
5	24
6	8
7	2

Finally, the updated transcriptome includes 3,868 completely novel genes, with 4,184 transcripts. In 2,277 of these genes, our annotation efforts identified at least one putative protein-coding sequence that was used for subsequent analysis, leaving 1,591 putative non-coding transcripts. Our S2 Fig presents one example of each type of update (novel transcript of an existing gene, novel gene that joins two or more previously defined genes, and completely novel GBIG genes). These Integrated Genome Viewer (IGV) representations of both annotations, as well as the reduced BAM alignment file, show the evidence supporting the updated annotations. The BAM file, and its associated BAI index, are available online:  https://doi.org/10.7910/DVN/2BIYNK.

We employed several filtering approaches and quality control measures to reduce our novel transcripts to a high-confidence set. In the initial StringTie construction of transcripts from the alignment of the reduced and joined BAM file, novel splice junctions required at least 4 reads with at least 11 bases aligned in each putative exon. Transcripts that passed these thresholds were integrated into an intermediate transcriptome (containing 66,362 transcripts, of which 33,380 were novel), and then for final filtering, all reads from all samples were aligned and quantified with STAR and RSEM (see Methods) against the intermediate transcriptome. In this final filter, transcripts that were not expressed in at least 30% of the initial samples, or that represented less than 10 percent of the expression of the transcript’s gene, or that had fewer than 10 summed reads across all samples were eliminated to generate the final transcriptome.

To confirm that novel transcripts were not disproportionately derived from repetitive or transposable element sequences, we compared the fraction of soft-masked sequence between GBI-annotated and novel transcripts. The novel transcripts showed a lower distribution as compared to the original (S3 Fig), indicating that repetitive element contamination was not a significant source of false positives in the novel genes or transcripts. Finally, further evidence supporting the validity of the novel annotations comes from read assignment data: novel transcripts were assigned substantial, and on average greater, read counts across samples (S Fig 4 and 5). This is especially true in the comparison of the novel transcripts that supported the joining together of previously separate GBI annotated genes. This provides strong evidence that these novel annotations represent genuinely expressed transcripts rather than assembly artefacts.

**Fig 1:**
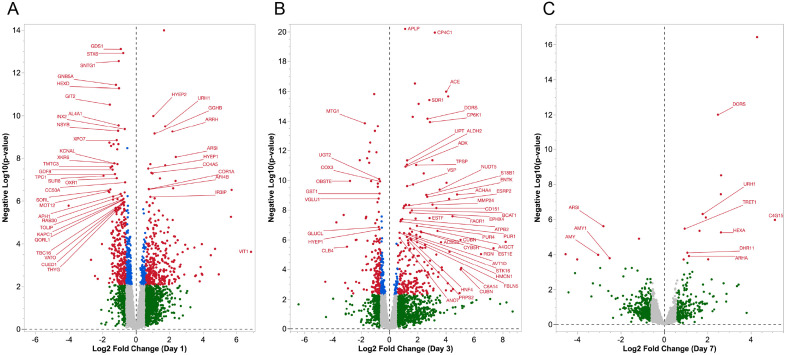
Volcano plots of differential gene expression in *G. bimaculatus* prothoracic ganglia at (A) one day, (B) 3 days, and (C) 7 days after deafferentation. Red dots represent genes that were determined to be differentially regulated by DESeq2, based on an absolute value of log2 fold change greater than 0.6 and an adjusted p-value less than 0.1. For visualization, all p-values less than 10^−10^ were set to 10^−10^. Blue dots show genes that were above threshold for adjusted p-value, but not log2 fold change. Green dots indicate genes that were above threshold for log2 fold change, but not for the adjusted p-value threshold. Note that the vertical plot axis is based on the p-value while the threshold for significance is based on the adjusted p value (FDR).

**Fig 2:**
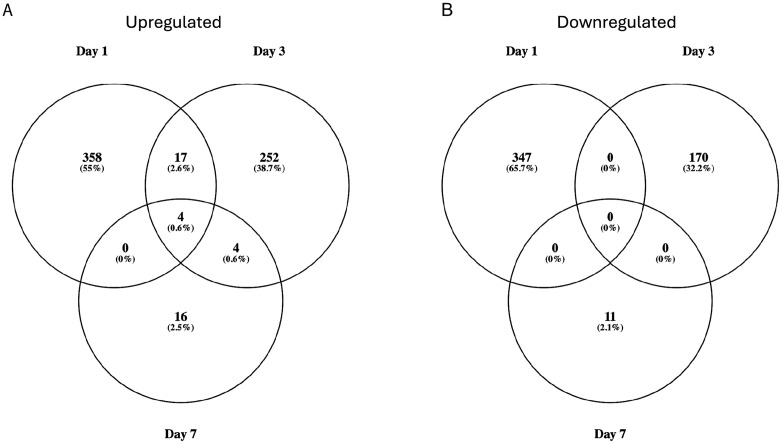
Venn diagrams show the total number (and percentage) of (A) upregulated genes and (B) downregulated genes across and between the three time points–one, three and seven days post-deafferentation. Lowly expressed genes were included. A small subset of genes were upregulated at multiple timepoints whereas downregulated genes showed no overlap across timepoints.

**Fig 3:**
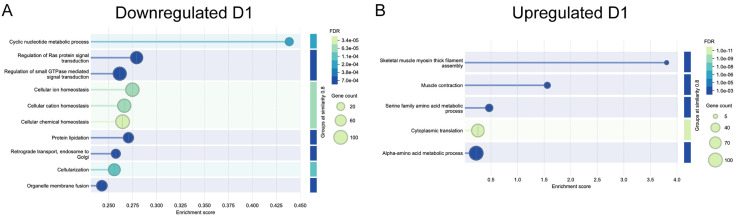
Visualization of functional enrichment of gene ontology biological process terms identified as significantly (A) downregulated and (B) upregulated in the prothoracic ganglia one day after deafferentation. Each bubble represents a representative GO term from a cluster of semantically related terms. Bubble size reflects the number of genes associated with each term, and bubble color indicates the false discovery rate (FDR), with darker blue corresponding to greater significance. The x-axis shows the enrichment score for each term.

BUSCO (Benchmarking Universal Single-Copy Orthologs) analysis [19,20] is a standard approach for assessing the completeness of a genome or transcriptome, based on conserved protein-coding orthologs. Using evolutionarily informed expectations of gene content, BUSCO analysis assesses the presence and multiplicity of genes that have been identified to be near-universally present as single-copy across defined phylogenetic ranges. The BUSCO scores for our updated transcriptome indicate a high quality assembly (Table 3), which improved upon the reference assembly [15]. Using BUSCO version 5.7.0 and the arthropoda_odb10 reference, the number of missing genes in the updated transcriptome was reduced from 26 to 7 and the number of fragmented genes from 22 to 5.

**Table 3:** BUSCO scores for the current assembly as compared to the annotations for the recent assembly of the genome.

	Genome Annotation		Current Assembly	
**Arthropoda:**	**Count**	**%**	**Count**	**%**
complete- total	965	95.26	1001	98.82
complete-single	916	90.42	945	93.28
complete-duplicated	49	4.84	56	5.53
fragmented	22	2.17	5	0.49
missing	26	2.57	7	0.69
total	1013		1013	

It is noteworthy that in the case of five BUSCO genes that were missing in the GBI assembly but present in our updated assembly, inspection showed that the newly identified matches were to genes that were present in the GBI transcriptome, however, the BUSCO matches were only identified with the addition of transcript isoforms in our new GBIG transcriptome (S2 Fig).

### Differential expression during compensatory plasticity

Pairwise comparisons of normalized counts data from deafferented *vs*. control crickets were performed at each time point using the R package DESeq2 (https://doi.org/10.7910/DVN/2BIYNK) using a False Discovery Rate (FDR) threshold of less than 0.1 and an absolute value threshold for log2 fold changes of greater than 0.6. The distribution of differentially expressed genes was visualized using volcano plots (Fig 1). Of the 726 genes identified as differentially expressed at 1-day post-deafferentation, only about 3.5% were downregulated 4-fold or more (12/347), while 17% were upregulated 4-fold or more (63/379). The remaining majority (50.1%) of significantly regulated genes at this early time point were upregulated more moderately at 2–4-fold. The remaining downregulated genes were fairly evenly split between 2–4-fold downregulated (48.1%) or less (48.4%; Fig 1A). Three days after deafferentation, 11.8% (20/170) were downregulated 4-fold or greater, 24% were downregulated 2–4-fold (41/170), while 64% (109/170) were downregulated less than 2-fold. For upregulated genes, 46.8% (118/252) of genes were upregulated 4-fold or greater (Fig 1B). At seven days a relatively low number of genes were identified as differentially regulated, but the fold-changes were large. Over 90% (10/11) were downregulated 4-fold or greater. More genes were upregulated rather than downregulated at seven days, but only 70.8% (17/24) of these were upregulated 4-fold or greater (Fig 1C).

We used Venn diagrams to explore how many genes were differentially regulated acrossmultiple days (Fig 2). The largest set of genes was upregulated at one day post-deafferentation (379), and 17 genes were uniquely shared between day one and day three (Fig 2A). Just over half of these 17 genes were unknown or uncharacterized genes (9/17); the genes that did have BLAST hits in this group included Beta-glucuronidase (GBI_01953), Putative accessory gland protein (GBI_04951), Dihydrofolate reductase (GBI_02419), Angiotensin-converting enzyme (GBI_15792), the Toll receptor Tollo (GBI_15807), Farnesol dehydrogenase (GBI_19142), and Acetylcholinesterase (GBIG_G_009355). Of the 24 genes upregulated at seven days, four genes were uniquely upregulated at day three and day seven, including two different Hexamerins (GBI_14215, GBI_14213). Four genes were upregulated across all three time points. Two of these genes were identified, including Embryonic polarity protein dorsal (GBI_10428) and the Putative URH1 protein (GBI_16625). The other two matched a protein of unknown function (GBI_16129) or did not have a protein match in NCBI (GBI_16644; Fig 2A). When we examined the downregulated genes, we found the most to be downregulated on day 1 (347 genes) and that none were shared across multiple days (Fig 2B).

After removing lowly expressed genes (mean count >70), the ten transcripts with the largest fold-changes at each time point (except for those downregulated at seven days, for which there were only eight), revealed that about half (51%) were unidentified (Table 4). Of these 30 unidentified genes, 43.3% were proteins of unknown function, while 56.7% showed no significant similarity to any proteins in the NCBI database (determined by a 1e-10 threshold in BLAST-x), though only three genes appear to have no open reading frames (“NA”). Genes lacking open reading frames and showing no significant protein similarity are candidate lncRNAs, which are increasingly recognized as important regulators of gene expression in neuronal tissues [16,17].

**Table 4:** Top 10 upregulated and downregulated candidates by day after deafferentation. After further filtering out lowly expressed genes (<70), the highest-fold change genes are reported along with predicted protein length (for the longest isoforms if relevant), log fold-change, adjusted p-value and BLAST-x results. The BLAST E-value threshold for proteins deemed to have no match was > 1e-10, and those without a predicted open reading frame were noted as NA.

Genome Assembly ID (>70)	Predicted Protein Length	Log Fold-Change	Adjusted p-Value	BLAST-x Result (gene name if available)
Day 1 Upregulated				
GBI_08969	106	7.342973839	9.20E-07	Protein of unknown function
GBI_19006	2,127	6.86252524	0.010159994	Vitellogenin-1 (VIT1)
GBI_00345	233	5.706071179	8.19982E-05	Protein of unknown function
GBI_04026	123	5.667125494	0.00068614	No protein match found
GBI_09392	68	4.939803059	0.050620643	Protein of unknown function
GBIG_G_007918	NA	4.718356724	0.008051719	No protein match found
GBI_21898	169	4.469353797	0.013077963	Myosin heavy chain2C muscle (MYSA)
GBI_22177	246	3.961636099	0.052231513	Actin-87E (ACT1)
GBI_09921	526	3.932166827	0.005834107	Putative MAL2 protein (MAL2)
GBI_00028	445	3.921908349	0.048581402	Actin-related protein 5 (ACT)
Day 1 Downregulated				
GBI_17240	707	−9.902407355	5.25E-14	No protein match found
GBI_14155	185	−4.020930638	0.00022171	Protein of unknown function
GBI_03017	761	−2.924148919	0.08408005	Toll-like receptor Tollo (TLR2)
GBIG_G_012940	57	−2.772674646	0.08408005	No protein match found
GBI_18011	243	−2.686600759	0.04822576	Protein of unknown function
GBIG_G_004248	49	−2.498522751	0.06311496	No protein match found
GBIG_G_009011	69	−2.454473866	0.00541163	No protein match found
GBI_13795	1,747	−2.290998946	0.00434769	Probable chitinase 10 (CHI10)
GBI_16320	543	−2.164857155	3.46E-06	Protein wntless (WLS)
GBI_07571	698	−2.038832442	0.07130012	Menin (MEN1)
Day 3 Upregulated				
GBI_02618	615	9.456788138	1.18974E-05	Protein lines (LINES)
GBI_14312	577	8.217629523	0.000154039	No protein match found
GBI_09392	68	7.358794362	0.000361759	Protein of unknown function
GBIG_G_008848	32	6.554286429	3.74E-07	No protein match found
GBI_10707	334	6.477214039	0.000773949	Regucalcin
GBI_03990	453	5.595465554	1.31E-08	No protein match found
GBIG_G_006626	525	5.08227045	0.004774592	Putative C6A14 protein (C6A14)
GBIG_G_000522	27	5.038965885	0.005725625	Putative CUBN protein (CUBN)
GBI_04847	2,965	5.030317122	0.000124019	Cubilin homolog (CUBN)
GBI_09641	576	4.957841121	0.086306628	Trehalase-like protein (TREA)
Day 3 Downregulated				
GBI_16413	57	−4.526013353	0.025631551	Protein of unknown function
GBIG_G_015481	500	−3.742745719	1.21719E-05	Protein of unknown function
GBI_01305	197	−3.609035013	0.099754226	Larval cuticle protein 1 (CUD4)
GBI_13799	66	−3.250954942	0.00145093	Protein of unknown function
GBIG_G_001703	73	−3.228212031	4.83E-06	No protein match found
GBI_14967	57	−3.119604757	0.028620374	Protein of unknown function
GBI_03346	814	−2.994772652	0.000295566	Serine protease grass (CLB4)
GBIG_G_008892	81	−2.927844222	0.052790643	No protein match found
GBI_11814	220	−2.779534253	4.70E-08	Protein obstructor-E (OBSTE)
GBIG_G_001080	66	−2.647115196	0.069444498	No protein match found
Day 7 Upregulated				
GBI_15110	466	6.163141223	0.071422913	Protein of unknown function
GBI_08082	541	5.112014845	0.001317578	Cytochrome P450 4g15 (C4G15)
GBI_03990	453	4.733021786	2.07E-27	No protein match found
GBI_15745	701	4.287076963	2.44E-13	No protein match found
GBIG_G_002140	111	3.93208328	1.21E-08	No protein match found
GBI_14213	205	3.628681797	0.003896256	Hexamerin-like protein 2 (CRPI)
GBI_14215	264	3.383694371	0.008889402	Hexamerin-like protein (HEXA)
GBI_00444	229	2.638873512	0.001317578	Hemolymph juvenile hormone-binding protein
GBI_17962	154	2.625526602	7.88E-06	Ankyrin repeat domain-containing protein 27
GBI_21440	266	2.621011068	0.004212023	Putative HEXA protein (HEXA)
Day 7 Downregulated				
GBIG_G_014995	NA	−4.552344733	0.047175527	No protein match found
GBIG_G_013309	959	−4.475913795	0.046302655	Putative POL4 protein (POL4)
GBIG_G_003038	NA	−4.017672381	0.071422913	No protein match found
GBI_10362	127	−3.756783999	0.009292717	Protein of unknown function
GBIG_G_015652	1,052	−3.049597897	0.049086473	Alpha-amylase-related protein (AMY)
GBI_11328	728	−2.809013134	0.002421449	Extracellular sulfatase SULF-1 homolog (ARSI)
GBI_02488	565	−2.519634262	0.071422913	Alpha-amylase 2 (AMY1)
GBI_01713	380	−1.167186913	0.008591585	Protein of unknown function

Of the genes with large-fold changes that could be identified, a few candidates were particularly notable. First, it was surprising to find such strong differential expression of several genes in this neuronal transcriptome that have not been previously associated with neurons, such as Vitellogenin (GBI_19006), Chitinase (GBI_13795), and Myosin heavy chain (GBI_21898). Vitellogenin is a lipid transport protein that functions as an egg yolk precursor protein, but is known to be expressed in glia in the central nervous system of honey bees [21] and likely regulates caste differentiation in those insects [22]. Chitinases may play a role in the support of the air-filled trachea [23], which branch through neuronal tissues. Chitinases also appear to have evolved a role in neuroinflammation in mammals and are currently being used as biomarkers for neurological disorders [24]. Myosin heavy chain protein is a muscle-related gene identified in this analysis that was upregulated more than 22-fold, part of a larger group of differentially expressed muscle-related genes that we discuss below.

Several candidates in Table 4, including Regucalcin (GBI_10707) and Alpha-amylase (GBI_02488), were identified by us in past suppression subtractive hybridization experiments [25]. Though at the time we proposed a role in immune defense, stress response, and energy metabolism, we now know that Alpha amylase functions to degrade glycogen within synapses and is important for normal synaptic function [26]. Regucalcin is important for calcium homeostasis and may protect against oxidative damage [27]. More recent results show that Regucalcin may also provide resistance to oxidative stress, as has been specifically shown for amyloid-β toxicity in PC12 cells [28].

The greater than 4-fold downregulation of two additional genes, Wntless (GBI_16320) and Tollo (GBI_03017), at one day post-deafferentation was intriguing. The protein Wntless controls dendritic self-avoidance in *D melanogaster* and *C. elegans* [29]. In the cricket auditory system, a rapid downregulation of Wntless could hypothetically alter the rules that typically guide dendrites and set the stage for the dendritic reorganization seen after deafferentation. Finally, GBI_03017, identified as Tollo (Toll-8), is likely a Toll receptor. Tolls are receptors for the family of Spaetzle ligands. Toll receptors are most commonly associated with immunity and dorsal-ventral patterning in early development [30], but research in *D. melanogaster* suggests that Spz-Toll signaling may also have a neurotrophic-like role in neurons, regulating cell number, connectivity, and synaptogenesis [31].

### Protein-protein association network predictions

To gain a deeper understanding of the genes and predicted proteins identified in this analysis, we turned to STRING (Search Tool for the Retrieval of Interacting Genes/Proteins) analysis ([32]; see Methods), with a specific focus on GO Biological Process. We used the ranked-list analysis tool, sorting the DESeq2 output from each day on the basis of estimated log2 fold change, to identify functional groups of genes that were up- and/or down-regulated (https://doi.org/10.7910/DVN/2BIYNK). The suggestion is that coordinated and biased expression of a functionally related set of genes indicates a systematic activation, deactivation, or regulation of the related processes. Visualizing the protein networks in our data that were systematically regulated can help suggest new hypotheses for the molecular basis of the anatomical plasticity seen in the cricket.

Based on this STRING analysis, we saw the enrichment of dozens of groups of functionally related genes on day one, with the top several focused on signaling, membrane dynamics, metabolism, and homeostasis (Fig 3). The enrichment of GTPase regulatory processes among downregulated genes was especially intriguing, because GTPases are central to cytoskeletal rearrangements and because morphological changes in dendrites are evident only a few days post-deafferentation (Fig 3A).

**Fig 4:**
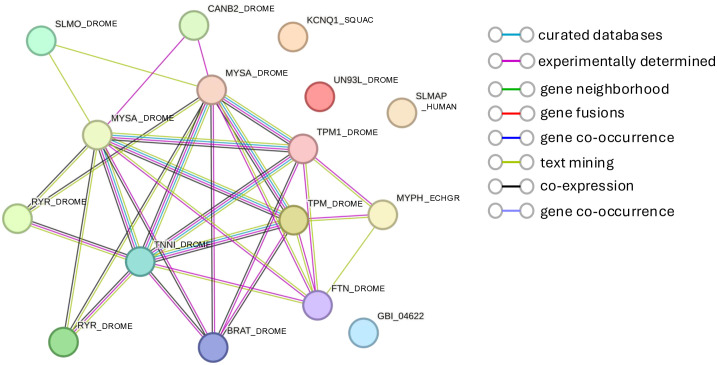
STRING diagram representing the protein association pathway derived from 16 muscle-related genes identified as enriched at one day post-deafferentation in our neuronal transcriptome. The average local clustering coefficient as reported by STRING was 0.362 and a PPI enrichment p-value was < 1.0e-16. Known associations from curated databases are shown in turquoise and those experimentally determined are shown in pink. Predicted associations are shown in green (gene neighborhood), red (gene fusions), and purple (gene co-occurrence). Additional associations are predicted from text mining (light green), co-expression (black), and protein homology (light purple).

Given that we isolated exclusively prothoracic neuronal tissue for this transcriptome, one of the biggest surprises was the enrichment of muscle-related genes upregulated one day after deafferentation (Fig 3B), specifically in the clustered GO terms “Skeletal muscle myosin thick filament assembly,” and “muscle contraction” (Fig 3B). STRING uses the term “interaction” to refer to any statistically significant associations between and among proteins, such as co-expression or co-occurrence in manuscripts. Where possible, we refer to this as an “association,” though we rely on their “protein-protein interaction” (PPI) values. Sixteen different transcripts, typically thought to be expressed predominantly or even exclusively in muscle, were enriched in our adult prothoracic ganglia after deafferentation, showing a PPI enrichment p-value of <1.0e-16 (Fig 4).

Several of these proteins are thought to be exclusively expressed in muscle. For example Flightin (FTN; GBI_10829), first discovered in *D. melanogaster* flight muscles, was shown with immunoblots to be absent from other tissues [33]. On the other hand, many other proteins initially characterized in muscle have clearly been identified in neurons in other species, and several have important roles in growing neurons. For example, there is some limited evidence that Troponin (TNNI; GBI_04010; [34]), Tropomyosin (TPM1; GBI_11216; [35]) and isoforms of Myosin heavy chain proteins (MYSA; GBI_13123; GBI_21898; [36]) are expressed in neurons, and that they can function there to modulate neuronal morphology, including in the advancement and turning of growth cones [35,36]. Our results raise the possibility that this set of proteins may have important roles to play in neurons, and possibly in the anatomical plasticity of neurons, beyond their known functions in muscle development and proliferation.

**Fig 5:**
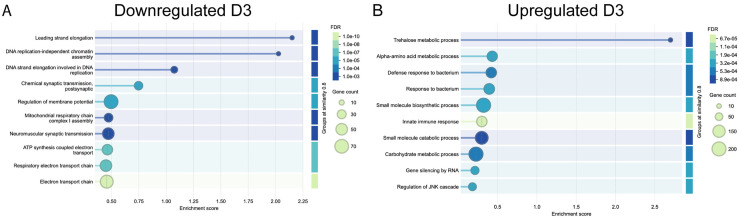
Visualization of functional enrichment of REVIGO TreeMap summary of gene ontology biological process terms identified as significantly (A) downregulated and (B) upregulated in the prothoracic ganglia three days after deafferentation. Each bubble represents a representative GO term from a cluster of semantically related terms. Bubble size reflects the number of genes associated with each term, and bubble color indicates the false discovery rate (FDR), with darker blue corresponding to greater significance. The x-axis shows the enrichment score for each term.

At three days post-deafferentation, based on the STRING analysis, it appears the deafferented neuronal tissue continues to adapt metabolically, while simultaneously engaging in stress response and cellular adaptation processes (Fig 5). Notably, several GO terms related to metabolic processes were enriched. The downregulation of proteins involved in synaptic signaling may reflect the ongoing dendritic reorganization that occurs around three days post-deafferentation. We know from time course studies that lateral ascending neuron dendrites are shortened while medial ascending neuron dendrites begin to sprout and extend across the midline, growing towards the contralateral axons [4]. Though we had attempted to control for general injury-induced transcriptional changes by amputating the tarsus in our age-matched control animals (presumably a less-intrusive injury) many of the enriched proteins may be general responses to the injury, as is evident in GO terms related to responses to bacteria.

Several limitations of this study should be noted. The transcriptome assembly relies exclusively on short-read (100 bp) Illumina sequencing, which, while filtered stringently to minimize false positives, is inherently limited in its ability to resolve complete isoform structures. Future work employing long-read sequencing technologies would substantially improve isoform-level resolution and allow more confident annotation of novel splice variants. Additionally, a proportion of the newly annotated genes, particularly those lacking open reading frames or significant similarity to known proteins, likely represent lncRNAs. While lncRNAs are increasingly recognized as important regulators of gene expression and neural plasticity, their identity and roles among the candidates identified here will require further experimental validation. Finally, the differential expression analysis was conducted with a small number of biological replicates (n = 3 per condition), necessitating a relaxed FDR threshold appropriate for hypothesis generation rather than definitive gene-level conclusions. Nevertheless, this updated, tissue-specific transcriptome provides a valuable resource for the cricket research community and generates concrete, testable hypotheses, for example with regards to Toll signaling and muscle-related gene networks, which can now be pursued to elucidate the mechanisms underlying deafferentation-induced anatomical plasticity. Finally, the updated transcriptome, combined with the transcript-isoform level expression patterns demonstrates the importance of considering the completeness of reference transcriptomes when analyzing highly specialized tissues or conditions.

## Methods

### Animals, injury, and library preparation

**Fig 6:**
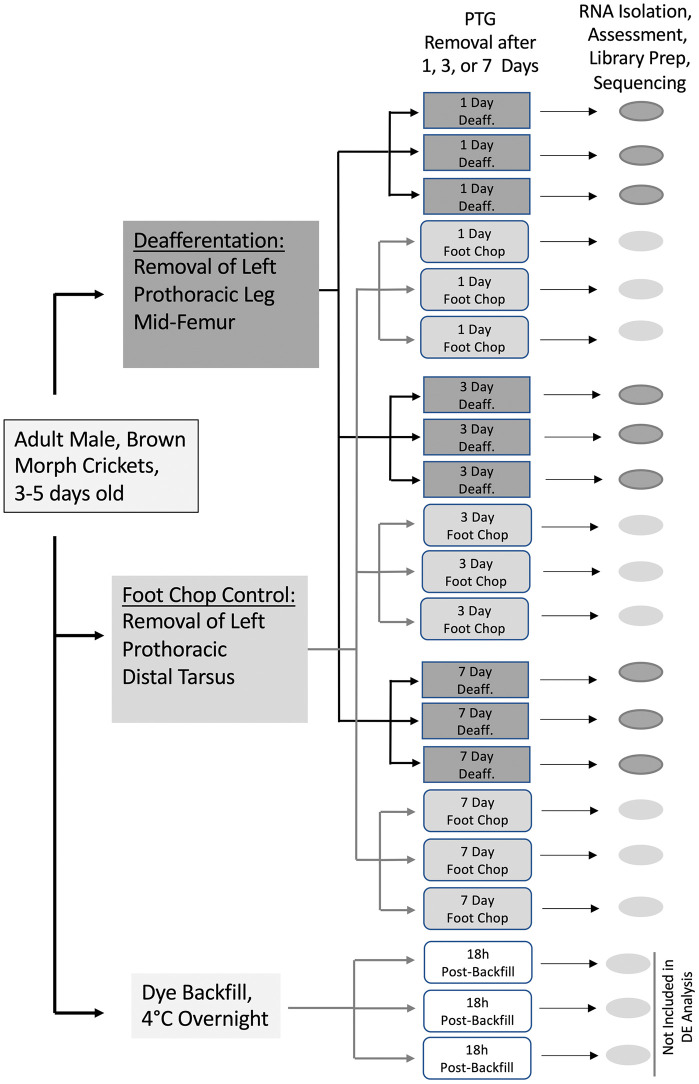
Summary of experimental design. 21 crickets, that were three to five days post adult eclosion, were amputated at the tarsal joint (“foot chop”) or mid-way along the femur (“deafferented”). Prothoracic ganglia were removed from deafferented or foot chop control animals one, three, or seven days post-injury. Three additional animals were backfilled 18 hrs prior to prothoracic ganglia removal; RNASeq data from these animals were included in the assembly but not in the differential expression.

Prothoracic ganglia from approximately 60 adult, male Mediterranean field crickets, *Gryllus bimaculatus,* were harvested and 21 individual ganglia were ultimately used as the sources of RNA for this transcriptome [12]. Male crickets that were adults for 3–5 days received either a control amputation of the distal segment of the left tarsus (“foot chop” control crickets), or the left prothoracic leg was severed mid-femur removing the auditory organ and deafferenting the ipsilateral central auditory neurons (“deafferented” experimental crickets). Males were chosen due to the potential sexual dimorphism in rates of dendritic growth after deafferentation [18]. Prothoracic ganglia were removed from crickets 1, 3, or 7 days after amputation at the femur or tarsus, or 18 hours post-backfill (Fig 6), and total RNA was purified as previously described [12]. In addition, several crickets were prepared for backfill as previously described [4]. This tissue was sequenced for a different experiment, was used for the assembly but excluded from the differential expression analysis.

The QIAGEN RNeasy Lipid Tissue Minikit was used to purify total RNA from each sample individually. RNA concentrations were assessed after TURBO DNA-free treatment (Ambion by Life Technologies) with a spectrophotometer (Nanodrop, Thermo Fisher Scientific) or a fluorometer (Qubit, Thermo Fisher Scientific). An Agilent 2100 Bioanalyzer (Applied Biosystems, Carlsbad, CA) was used to further assess sample quality. Based on evaluation of RNA quality and concentration of individual ganglion samples, the best 3 samples for each condition were selected for sequencing. Standard Illumina paired-end library protocols

were used to prepare samples. The Illumina Hiseq 2500 platform, running v4 chemistry to generate ~ 25M paired end reads of 100 bp in length for each sample, was used to sequence the RNA [12].

### Transcriptome analysis and update

The draft cricket genome (*G. bimaculatus*) and the associated annotation GTF file were downloaded from https://gbimaculatusgenome.rc.fas.harvard.edu/ in June 2022. We used a newly developed Nextflow pipeline, txome_refinement (https://github.com/mdibl/txome_refinement), that is designed to use empirical RNAseq data to update and revise a starting genome annotation [manuscript in preparation]. In brief, txome_refinement was derived from the Nextflow (https://nextflow.io/) NF-core (https://nf-co.re/) rnaseq workflow, version 3.8.1 (https://nf-co.re/rnaseq/3.8.1), and the initial stages of quality control and alignment of individual samples to the genome with the STAR aligner are identical. Alignment statistics for each sample can be found here: https://doi.org/10.7910/DVN/2BIYNK. Following alignment, each output BAM file was reduced using the program bamsifter, a utility that is part of the trinity rnaseq package [37], and then samtools merge [38] was used to join the reduced files into a single unified BAM, which was finally again reduced with bamsifter. The final aggregate BAM and the associated.BAM.BAI file (both available online: https://doi.org/10.7910/DVN/2BIYNK) were processed with StringtTe [39] to generate a sample-specific transcriptome. Critical parameters in the first StringTie run included -j = 4, requiring a minimum of 4 reads spanning any included splice junction, -a = 15, requiring at least 15 bases of anchor alignment for each included splice alignment, and --coverage = 10, setting the minimum coverage for inclusion in the output transcriptome. GFFcompare [40] was used to compare the novel sample-specific transcripts to the reference transcriptome. Finally, a novel program GTFinsert was used to join novel transcripts and the reference genome, resulting in a new intermediate GTF file describing the updated transcriptome. This intermediate GTF was then used as a target in a new alignment by STAR aligning all reads from all samples, followed by quantification with RSEM [41] for gene expression and RSEQC [42] for splice junction coverage. Novel transcripts were filtered out of the intermediate transcriptome if it failed any of three tests: (1) if the transcript had RSEM assigned expression in fewer than 30% of the input samples, (2) the overall number of sequence reads assigned to the transcript was fewer than 10, or (3) if the fraction of all sequence reads assigned to the transcript was less than 10 percent of all sequence reads assigned to the transcript’s gene. The novel transcripts that survived the filters described above were then reinserted into the original GBI annotation file as described above, resulting in the final reported transcriptome (available at: https://doi.org/10.7910/DVN/2BIYNK)

### BUSCO transcriptome analysis

BUSCO (Benchmarking Universal Single Copy Orthologs) assessment [43] of the transcriptome was performed using a Docker image of BUSCO version 5.7.0. Analyses were carried out against target set arthropoda_odb10 using the transcriptome analysis option. Final refinement was made by reconciling BUSCO matches from multiple transcript isoforms derived from a common gene resulting in the final “gene-level” tables presented here.

### Updated functional annotation of the GBIG transcriptome

In order to assess the likely functional roles of our updated transcriptome, we used the following approach: First, the “annotate only” option for the transpi workflow [44], which calls the trinotate pipeline, which is included in the trinityrnaseq package [45]. Trinotate assigns GO categories in three ways: (1) blastx [46] alignments of the predicted transcripts, (2) blastp [46] alignments of transdecoder-predicted proteins, and (3) pfam [47] matches identified with hmmer (http://hmmer.org). Second, the transdecoder-predicted protein-coding sequences were reduced to the longest protein for each gene (https://doi.org/10.7910/DVN/2BIYNK). Annotations were also available through the STRING interface [32], as described below. The transPi analysis produced at least one GO annotation for 11,211 genes, the STRING analysis produced at least one GO annotation for 11,955 genes. All functional annotations for the updated genome are available here: https://doi.org/10.7910/DVN/2BIYNK. The STRING-based tools for our updated transcriptome can be accessed at the STRING resource at URL: https://version-12-0.string-db.org/organism/STRG0A33TVI. Finally, any existing annotations in the reference GFF file were transferred directly to the new GTF.

### Differential gene expression analysis

Expression levels for each sample were generated for each original sample with the Nextflow Nf-core rnaseq pipeline (version 3.9) using the Salmon pseudo alignment only option, with the updated transcriptome definition provided to define target transcripts.

Gene counts from Salmon were analyzed for differential gene expression using the R package “DESeq2” (version 1.42.1; [48]), performing a pairwise comparison between deafferented and control at each time point, generating estimated log2-fold change, p-value, and adjusted p-value, according to the Benjamini–Hochberg method [49]. Genes were considered significantly up or down regulated if the False Discovery Rate (FDR) was less than 0.1 and the absolute value of the log2 fold change was greater than 0.6. Differentially regulated gene lists for each day were used to make a Venn diagram in Venny 2.1 (https://bioinfogp.cnb.csic.es/tools/venny/)

Examination of the principal components plots for Day 3 and Day 7 analysis resulted in the identification of samples that were clear outliers, therefore these samples were not included in the DESeq2 calculations. The design matrixes and count matrixes are included can be found online: https://doi.org/10.7910/DVN/2BIYNK.

### Functional enrichment analysis

To facilitate functional analysis, we used the “Annotated Proteome” feature, introduced in version 12 of the STRING database [50]. The STRING analysis first searches the uploaded proteome against its existing database to identify putative orthologs. STRING then transfers protein-protein association links from other organisms, based on several different types of evidence to assess associations, combining the relevant information into a single confidence score for each association. We used TransDecoder (https://github.com/TransDecoder/TransDecoder) to generate a putative proteome file for our updated transcriptome, then reduced to a single protein per gene locus by arbitrarily selecting the longest protein predicted for each gene across all transcript isoforms. Functional enrichment was carried out through the STRING database interface (https://string-db.org/), using the uploaded cricket proteome as the target organism (https://version-12-0.string-db.org/organism/STRG0A33TVI). Differential expression output from DESeq2 were threshold-selected to those with baseMean expression >= 100 counts and the resulting table of gene IDs and estimated log2 fold change were uploaded to STRING for use with the “Proteins with Ranks/Values” tool.

All searches of gene sets were carried out with default parameters. Gene Ontology Biological Process enrichment tables were downloaded from STRING and then segregated into up- and down-regulated terms. Enriched GO terms were visualized as bubble plots using STRING, with terms grouped at a semantic similarity threshold of 0.8. Each bubble represents a representative term from a cluster of semantically related GO terms, with bubble size proportional to the number of associated genes and color reflecting the false discovery rate.

## Supporting information

S1 Table List of genes with complete annotations, including both the new identifier along with the GBI genes that were joined. (XLSX)

S2 Fig Examples of updated GBIG transcriptome annotations. Examples of updated GBIG transcriptome annotations. In each panel, the sashimi plot shows the count of reads supporting each splice-junction, based on the reduced BAM file generated with our data. The annotation plots below show GBI annotations (in black if present) and GBIG annotations (in blue). (A) The joining of two neighboring genes in the GBI annotations (GBI_00289 and GBI_00290) are supported by multiple spliced alignments that span portions of both genes. (B) Our transcriptome data and annotation process adds a critical new transcript to annotated gene GBI_00895. The novel transcript provides a BUSCO match that was not identified based on the GBI annotations. The inset shows an expanded view of four novel exons that allow for this annotation. (C) GBIG gene GBIG_008456, identified on genomic Scaffold3, is a novel identification with support for 2 distinct transcript isoforms.
(PPTX)

S3 Fig Comparison of percentage soft-masked sequence in GBI and novel transcripts. The fraction of transcript length that is soft-masked plotted for either GBI or novel transcripts. The plots are limited to only transcripts that have non-zero soft-masked bases and the counts for each is shown in labels on the bottom.
(PDF)

S4 Fig Normalized expression distribution comparison between GBI and novel genes and transcripts. Normalized Expression by Transcript Type (transcripts per million): The genes are separated into “GBI” (where the gene was predicted to exist in the GBI annotation), “joined” (where 2 or more GBI genes were joined into a new gene), and “novel” (where there was no GBI annotation overlapping). Transcripts in the GBI and joined classes are separated into GBI and novel. There is, on average, significantly more support for the novel transcripts than for the GBI transcripts.
(PDF)

S5 Fig Raw expression distribution comparison between GBI and novel genes and transcripts. Plots the actual number of reads assigned to each transcript, for the original assembly and the current assembly. The genes are separated into “GBI” (where the gene was predicted to exist in the GBI annotation), “joined” (where 2 or more GBI genes were joined into a new gene), and “novel” (where there was no GBI annotation overlapping). There is, on average, significantly more support for the novel transcripts than for the GBI transcripts.
(PDF)
