# Supplementary material for: Integration of a neuronal RNAseq dataset with the draft Gryllus bimaculatus transcriptome refines gene predictions and highlights potential systematic response to injury
Source: PLoS One. 2026 Apr 29;21(4):e0347755. doi: 10.1371/journal.pone.0347755 (PMC13127959; doi:10.1371/journal.pone.0347755)
Supplement: S2 Fig — Examples of updated GBIG transcriptome annotations. In each panel, the sashimi plot shows the count of reads supporting each splice-junction, based on the reduced BAM file generated with our data. The annotation plots below show GBI annotations (in black if present) and GBIG annotations (in blue). (A) The joining of two neighboring genes in the GBI annotations (GBI_00289 and GBI_00290) are supported by multiple spliced alignments that span portions of both genes. (B) Our transcriptome data and annotation process adds a critical new transcript to annotated gene GBI_00895. The novel transcript provides a BUSCO match that was not identified based on the GBI annotations. The inset shows an expanded view of four novel exons that allow for this annotation. (C) GBIG gene GBIG_008456, identified on genomic Scaffold3, is a novel identification with support for 2 distinct transcript isoforms. (PPTX) [file pone.0347755.s002.pptx]

## Slide 1
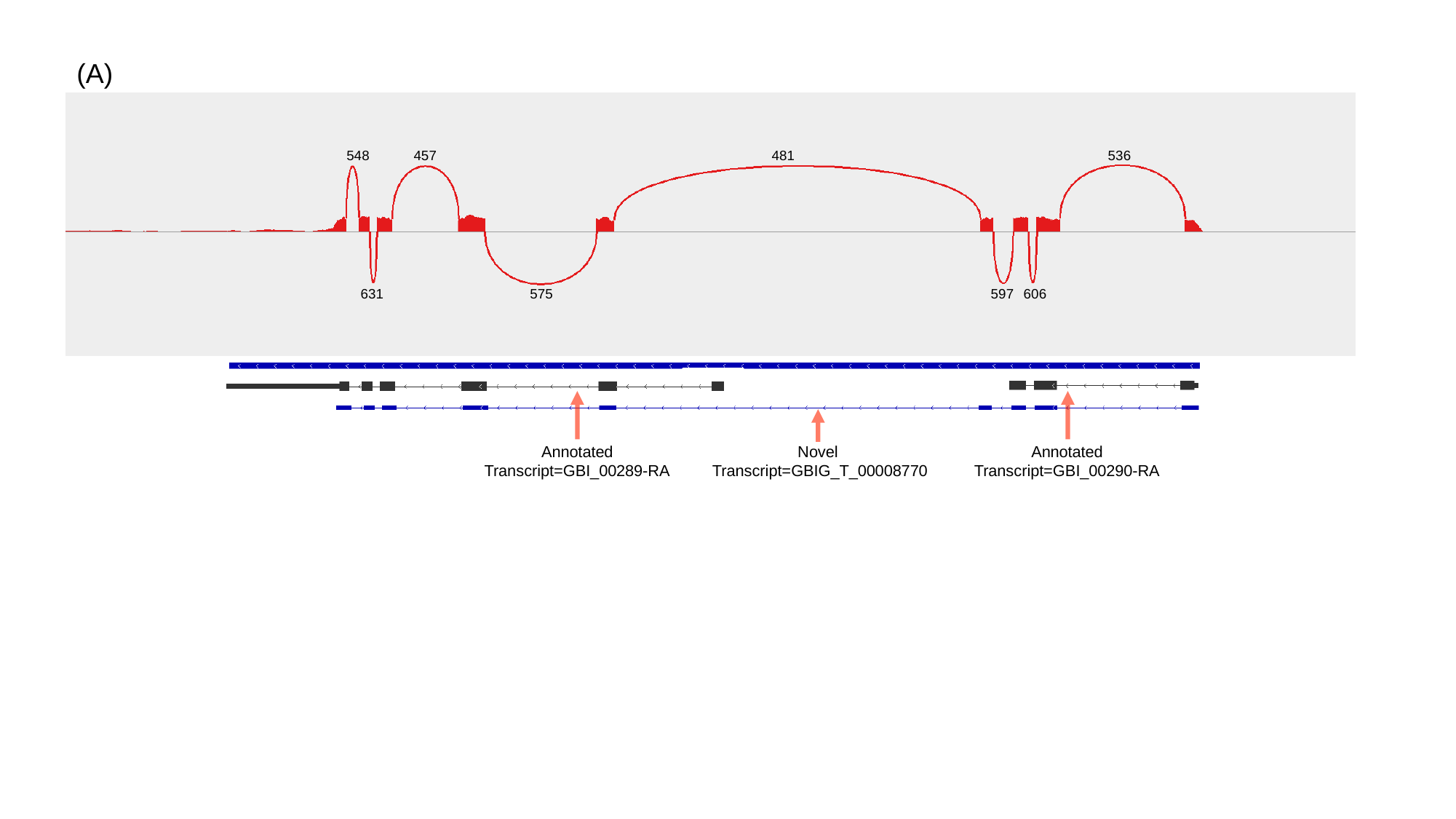

(A)
548
457
481
536
631
575
597
606
Annotated
Transcript=GBI_00289-RA
Novel
Transcript=GBIG_T_00008770
Annotated
Transcript=GBI_00290-RA

## Slide 2
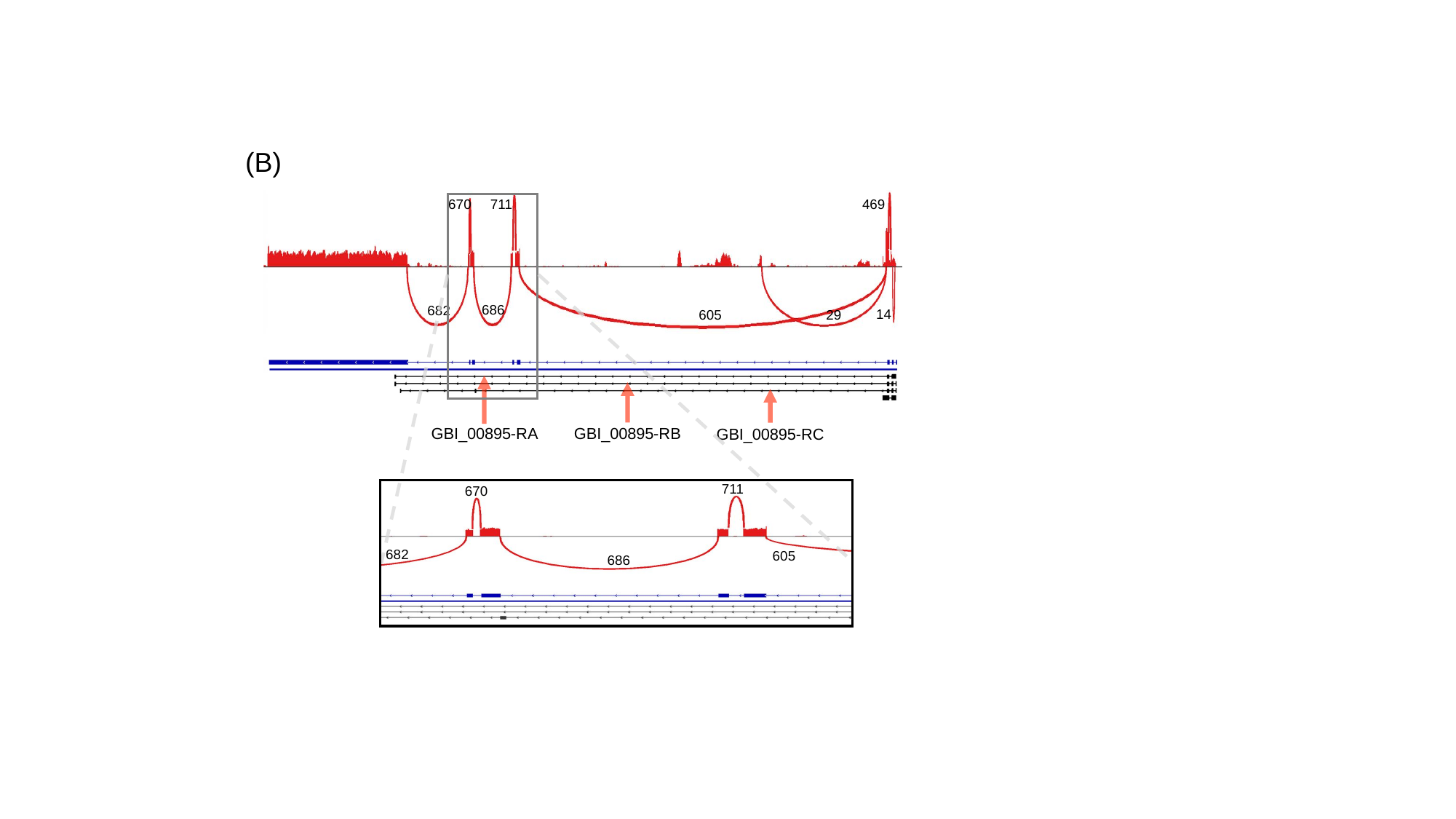

(B)
670
711
469
686
682
14
605
29
GBI_00895-RA
GBI_00895-RB
GBI_00895-RC
711
670
682
605
686

## Slide 3
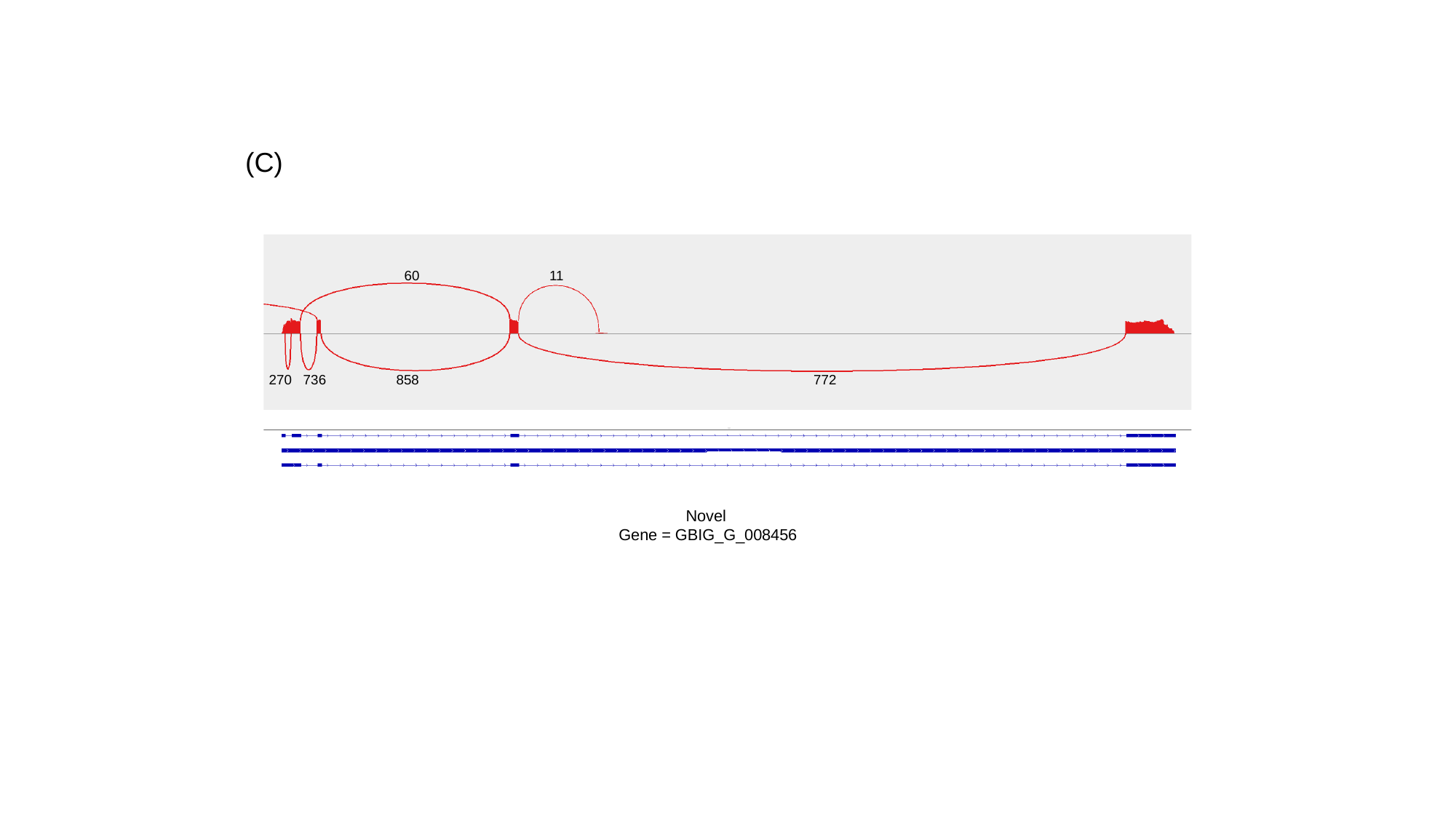

(C)
60
11
270
736
858
772
Novel
Gene = GBIG_G_008456

## Slide 4
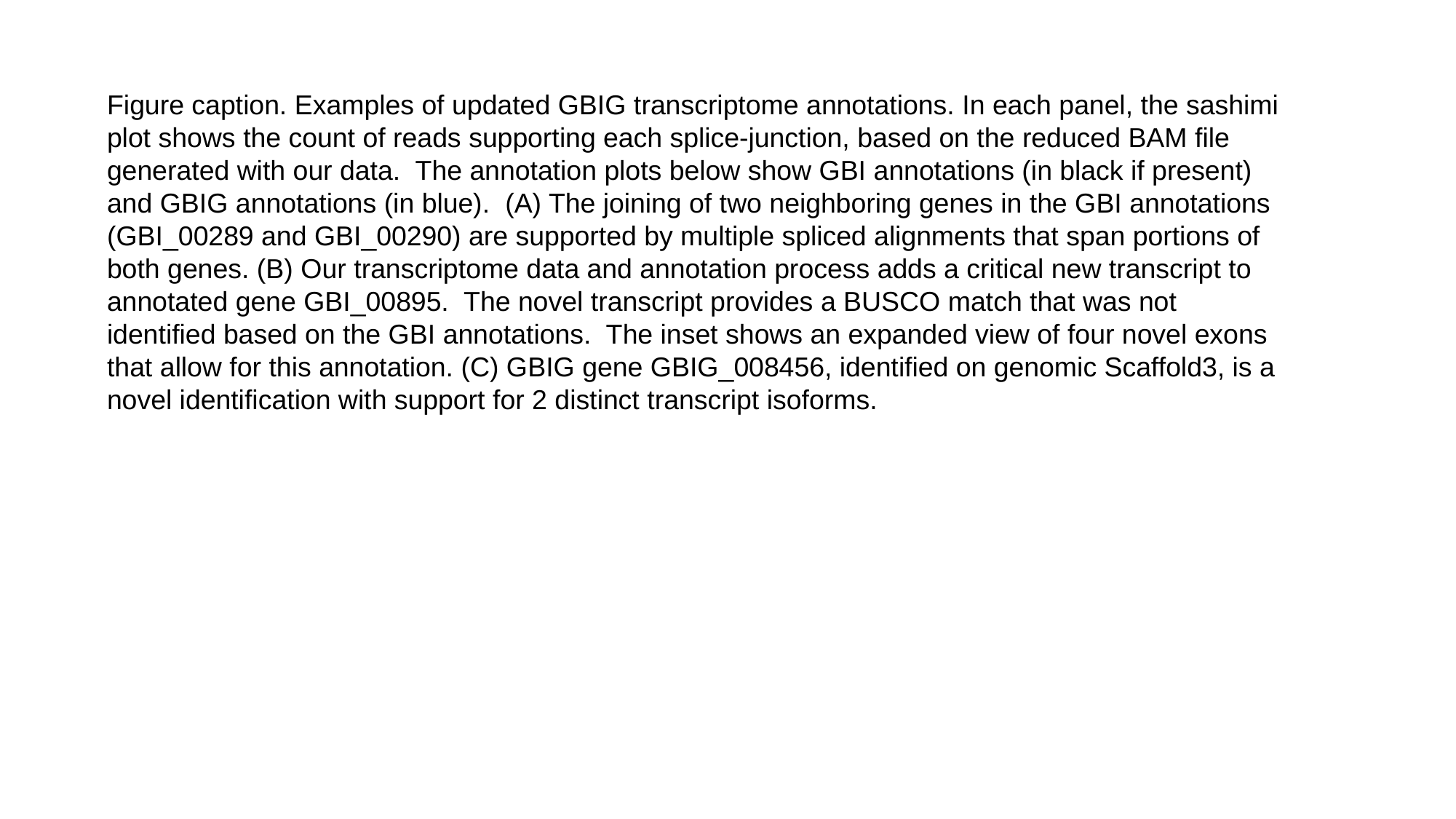

Figure caption. Examples of updated GBIG transcriptome annotations. In each panel, the sashimi plot shows the count of reads supporting each splice-junction, based on the reduced BAM file generated with our data. The annotation plots below show GBI annotations (in black if present) and GBIG annotations (in blue). (A) The joining of two neighboring genes in the GBI annotations (GBI_00289 and GBI_00290) are supported by multiple spliced alignments that span portions of both genes. (B) Our transcriptome data and annotation process adds a critical new transcript to annotated gene GBI_00895. The novel transcript provides a BUSCO match that was not identified based on the GBI annotations. The inset shows an expanded view of four novel exons that allow for this annotation. (C) GBIG gene GBIG_008456, identified on genomic Scaffold3, is a novel identification with support for 2 distinct transcript isoforms.
