# Supplementary material for: Integration of a neuronal RNAseq dataset with the draft Gryllus bimaculatus transcriptome refines gene predictions and highlights potential systematic response to injury
Source: PLoS One. 2026 Apr 29;21(4):e0347755. doi: 10.1371/journal.pone.0347755 (PMC13127959; doi:10.1371/journal.pone.0347755)
Supplement: S3 Fig — The fraction of transcript length that is soft-masked plotted for either GBI or novel transcripts. The plots are limited to only transcripts that have non-zero soft-masked bases and the counts for each is shown in labels on the bottom. (PDF) [file pone.0347755.s003.pdf]

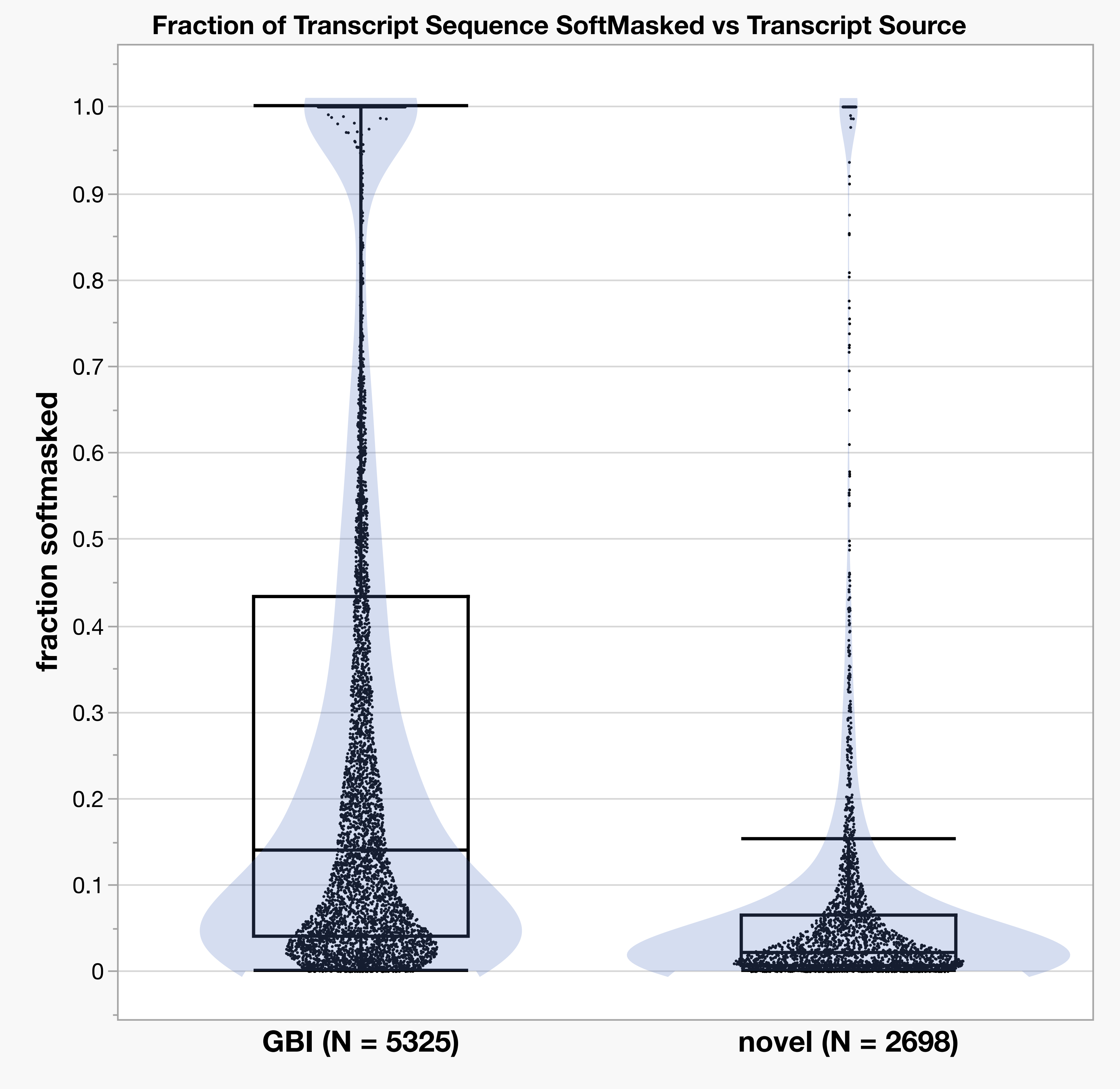
Fraction of Transcript Sequence SoftMasked vs Transcript Source
fraction softmasked
1.0
0.9
0.8
0.7
0.6
0.5
0.4
0.3
0.2
0.1
0
GBI (N = 5325)
novel (N = 2698)
