# Supplementary figures and images for: Integration of a neuronal RNAseq dataset with the draft Gryllus bimaculatus transcriptome refines gene predictions and highlights potential systematic response to injury
Source: PLoS One. 2026 Apr 29;21(4):e0347755. doi: 10.1371/journal.pone.0347755 (PMC13127959; doi:10.1371/journal.pone.0347755)

Distribution of Normalized Expression as a Function of Gene/Transcript Type

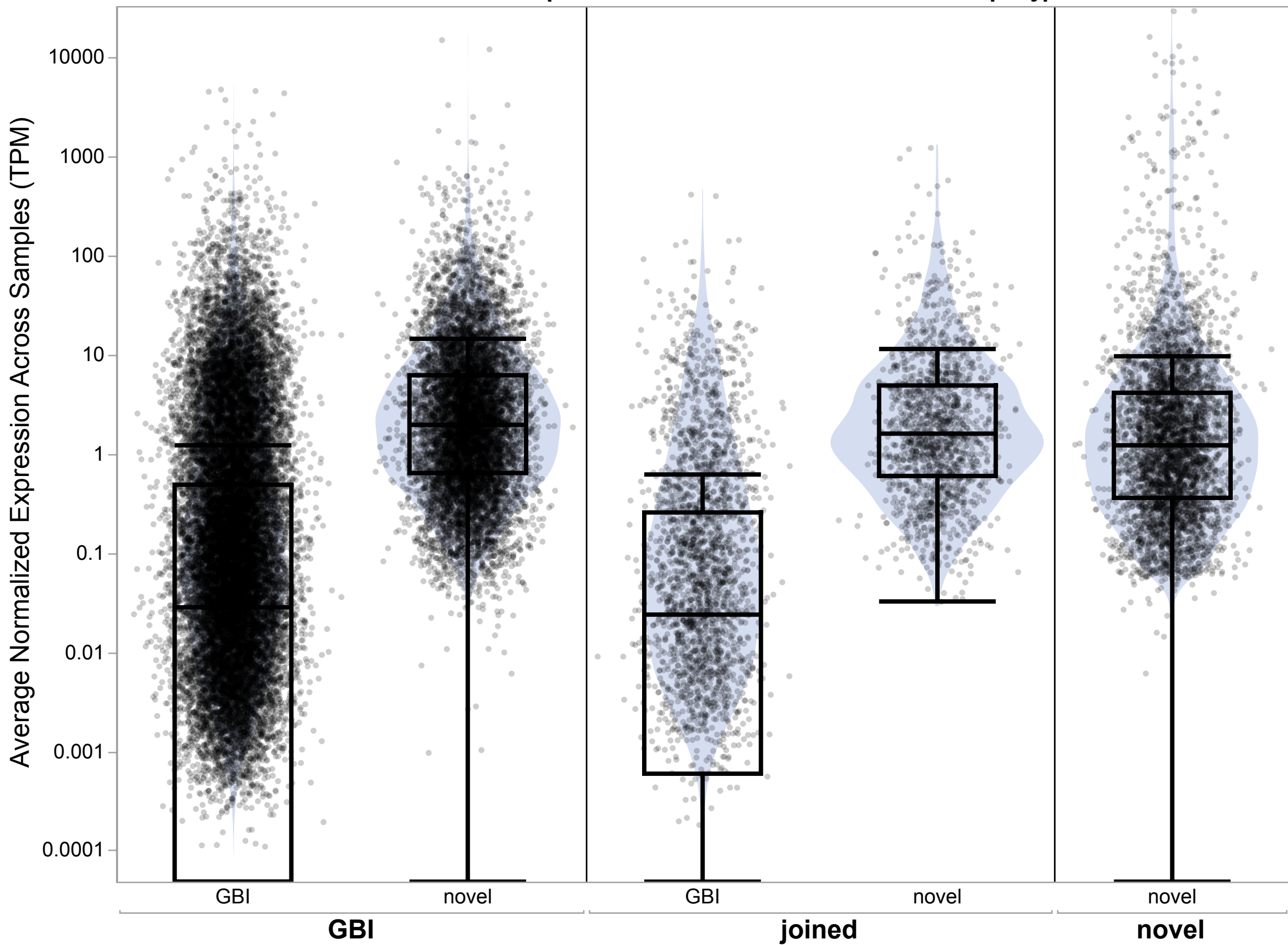

Supplement: S4 Fig — Normalized Expression by Transcript Type (transcripts per million): The genes are separated into “GBI” (where the gene was predicted to exist in the GBI annotation), “joined” (where 2 or more GBI genes were joined into a new gene), and “novel” (where there was no GBI annotation overlapping). Transcripts in the GBI and joined classes are separated into GBI and novel. There is, on average, significantly more support for the novel transcripts than for the GBI transcripts. (PDF) [file pone.0347755.s004.pdf]

Distribution of Assigned Expression as a Function of Gene/Transcript Type

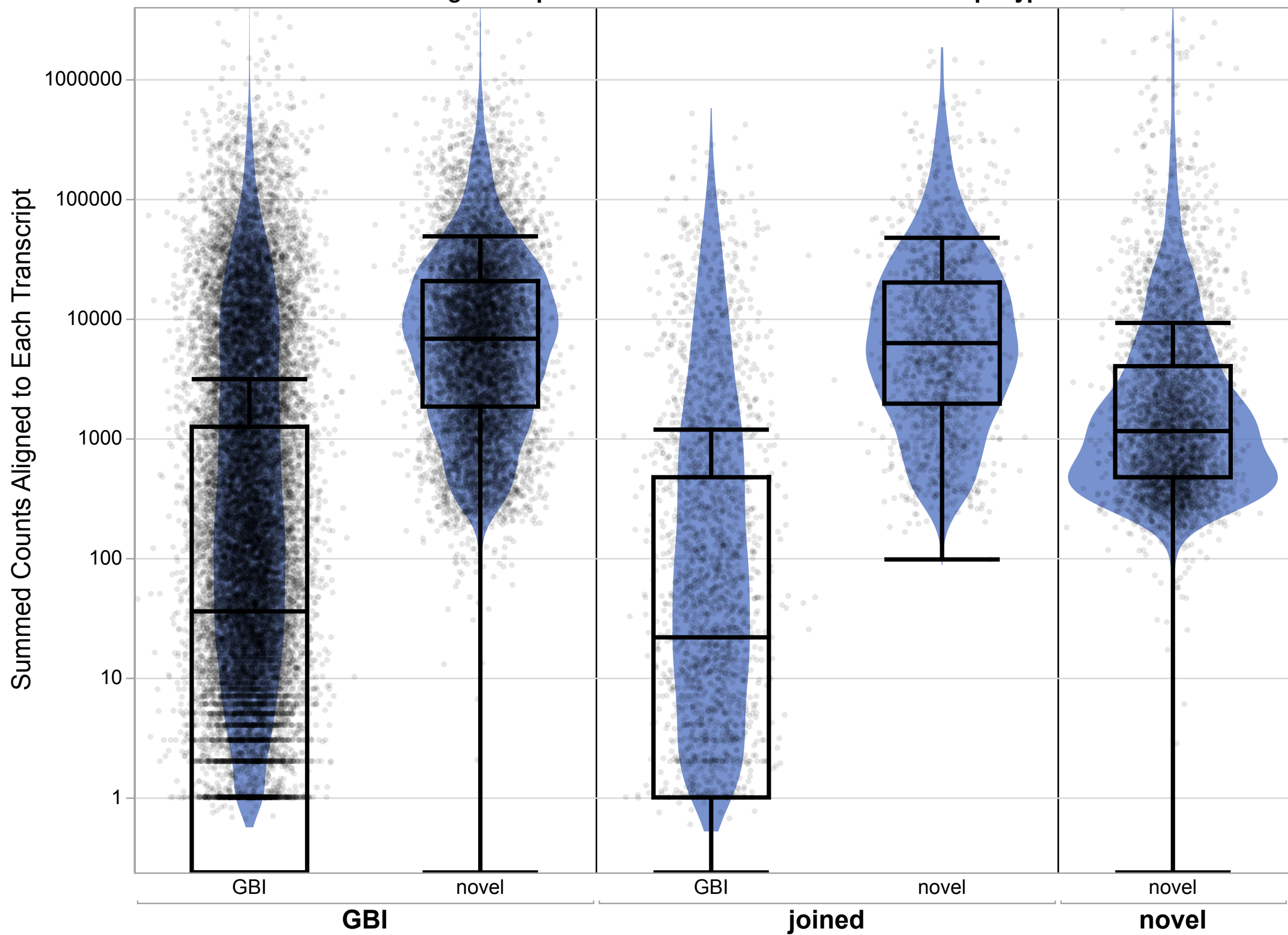

Supplement: S5 Fig — Plots the actual number of reads assigned to each transcript, for the original assembly and the current assembly. The genes are separated into “GBI” (where the gene was predicted to exist in the GBI annotation), “joined” (where 2 or more GBI genes were joined into a new gene), and “novel” (where there was no GBI annotation overlapping). There is, on average, significantly more support for the novel transcripts than for the GBI transcripts. (PDF) [file pone.0347755.s005.pdf]
